# Correction: Wang et al. CeO_2_-Supported TiO_2_−Pt Nanorod Composites as Efficient Catalysts for CO Oxidation. *Molecules* 2023, *28*, 1867

**DOI:** 10.3390/molecules29214977

**Published:** 2024-10-22

**Authors:** Haiyang Wang, Ruijuan Yao, Ruiyin Zhang, Hao Ma, Jianjing Gao, Miaomiao Liang, Yuzhen Zhao, Zongcheng Miao

**Affiliations:** 1 Xi’an Key Laboratory of Advanced Photo-Electronics Materials and Energy Conversion Device, School of Electronic Information, Xijing University, Xi’an 710123, China; 2 School of Materials Science and Engineering, Xi’an Polytechnic University, Xi’an 710048, China; 3 School of Artificial Intelligence, Optics and Electronics (iOPEN), Northwestern Polytechnical University, Xi’an 710072, China

Following publication, concerns were raised regarding the peer-review process related to the publication of this article [1]. Adhering to our standard procedure, the Editorial Board conducted an investigation which determined that, while the peer-review process does comply with MDPI’s Editorial Process policy (https://www.mdpi.com/editorial_process), the contribution of one of the four reviewers does not comply with MDPI’s Guideline for Reviewers (https://www.mdpi.com/reviewers#_bookmark11) nor the expectations of the Editorial Board. As a result, the Editorial Board has decided to remove the contribution of one of the reviewers from the open peer-review record (https://www.mdpi.com/1420-3049/28/4/1867/review_report) and, following discussion with the authors, have removed the citations (references [4,5] in the original publication) recommended by this reviewer from this publication [1]. The Editorial Board has confirmed that the publication of this paper in its modified form is in line with their expectations and MDPI’s Editorial Process policy. The authors state that the scientific conclusions are unaffected. The publication and associated webpage have been updated accordingly.
