# Supplementary material for: CeO2-Supported TiO2−Pt Nanorod Composites as Efficient Catalysts for CO Oxidation
Source: Molecules. 2023 Feb 16;28(4):1867. doi: 10.3390/molecules28041867 (PMC9959209; doi:10.3390/molecules28041867)
Supplement: Supplementary file 1 [file molecules-28-01867-s001.zip › molecules-2198344-supplementary.pdf]

Supporting Information

Enhanced catalytic performance of CeO_2 supported TiO_2-Pt nanorod in CO oxidation

Haiyang Wang [1,†], Ruijuan Yao [1,†], Ruiyin Zhang [1], Hao Ma [1], Jianjing Gao [1], Miaomiao Liang [2], Yuzhen Zhao [1], Zongcheng Miao [1,3,*]

[1] Xi’an Key Laboratory of Advanced Photo-electronics Materials and Energy Conversion Device, Key Laboratory of Organic Polymer Photoelectric Materials, School of Electronic Information, Xijing University, Xi’an, 710123, China

[2] School of Materials Science and Engineering, Xi’an Polytechnic University, Xi’an, Shaanxi, 710048, China

[3] School of Artificial Intelligence, Optics and Electronics (iOPEN), Northwestern Polytechnical University, Xi'an, Shaanxi, 710072, China

[†] These authors contributed equally to this work.

[*] E-mail: miaozongcheng@nwpu.edu.cn

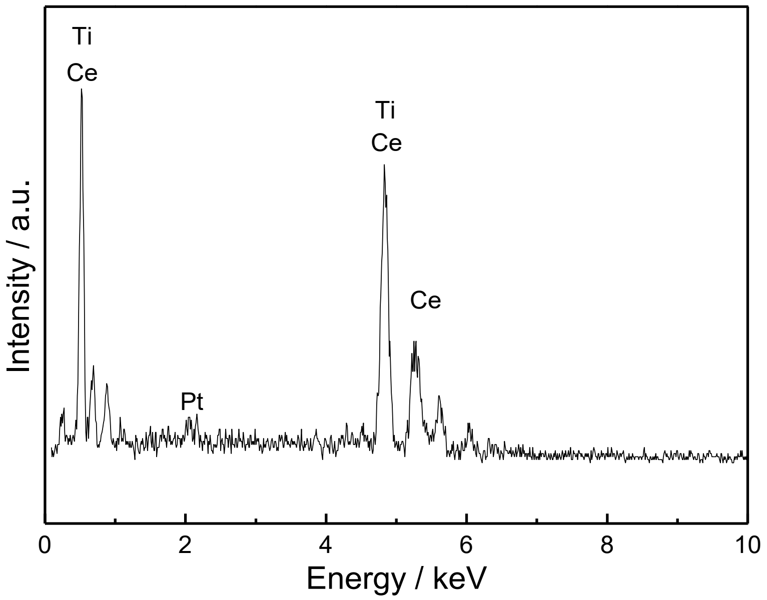

Figure S1 The EDS spectrum of $Al_{91.2}Ce_8Pt_{0.3}Ti_{0.5}$ melt-spun ribbons after dealloying and calcination treatment

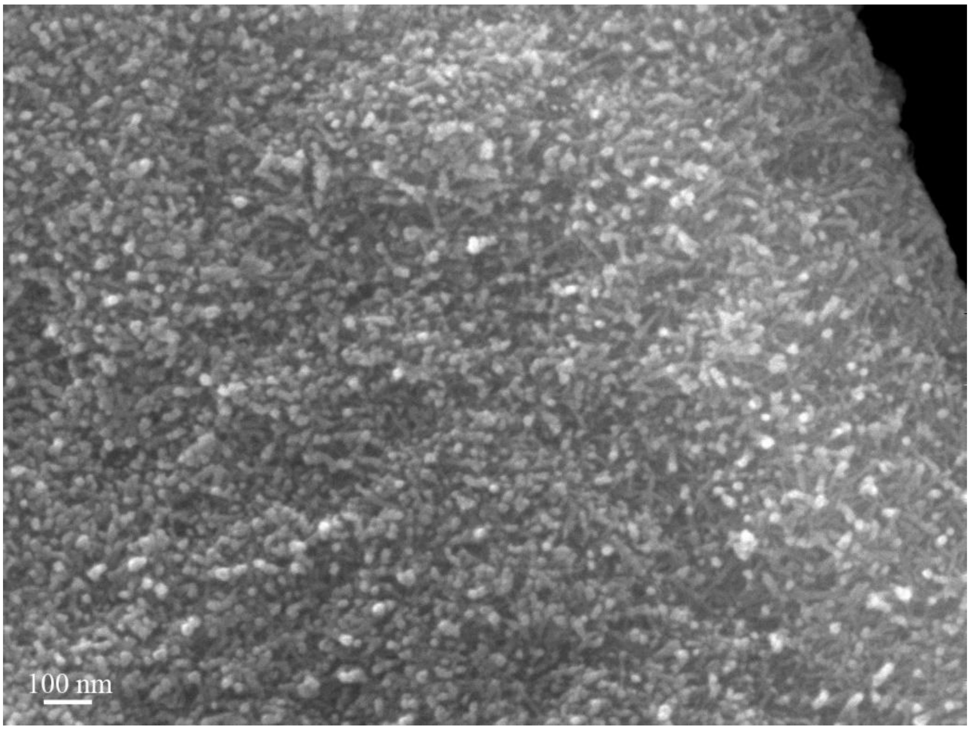

Figure S2 The cross-sectional SEM image of $(0.5TiO_2\text{-}Pt)/CeO_2$

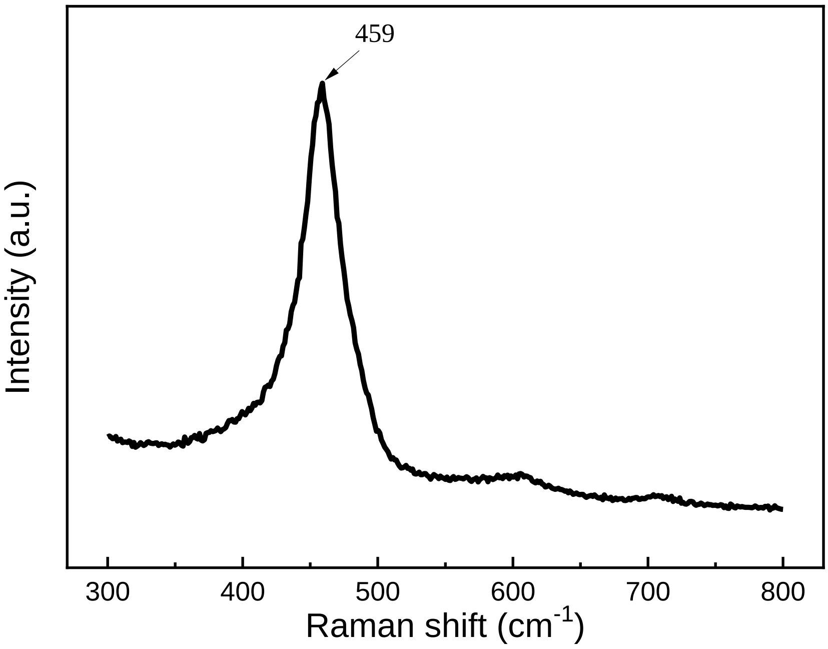

Figure S3 The Raman spectrum of pure CeO_2

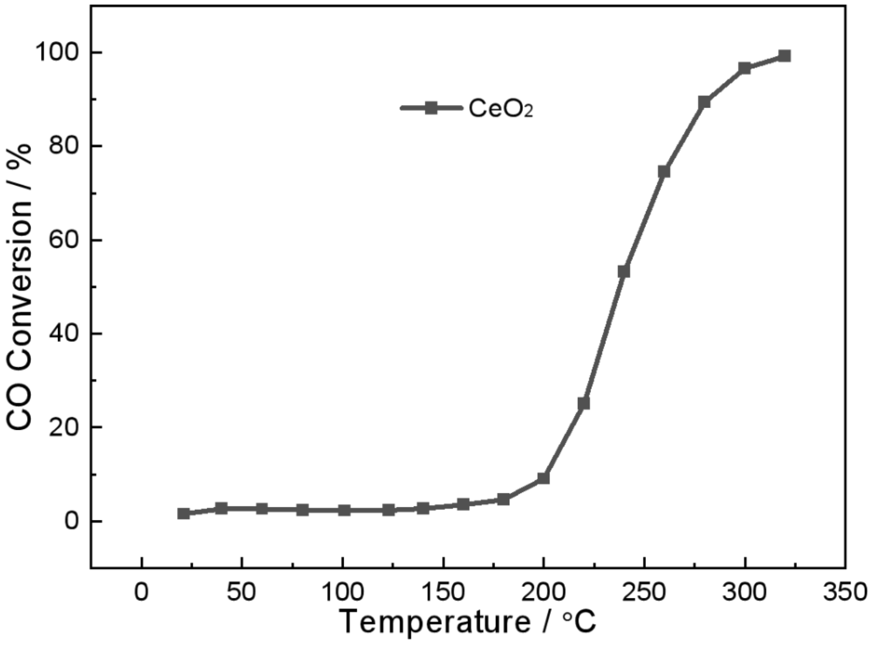

Figure S4 The catalytic performance of CeO_2 matrix

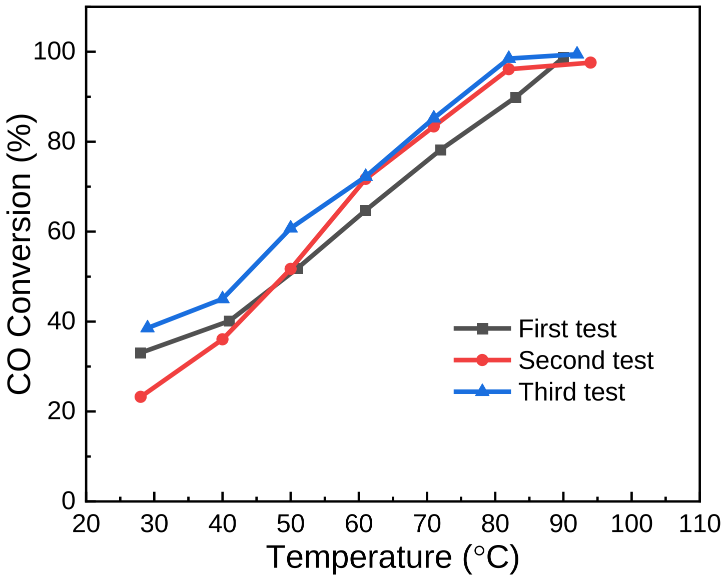

Figure S5 The reusability test of (0.5TiO_2-Pt)/CeO_2
